# Microenvironmental Factors that Shape Bacterial Metabolites in Inflammatory Bowel Disease

**DOI:** 10.3389/fcimb.2022.934619

**Published:** 2022-07-15

**Authors:** Lacey R. Lopez, Ju-Hyun Ahn, Tomaz Alves, Janelle C. Arthur

**Affiliations:** ^1^ Department of Microbiology and Immunology, The University of North Carolina at Chapel Hill, Chapel Hill, NC, United States; ^2^ Division of Comprehensive Oral Health, Adams School of Dentistry, The University of North Carolina at Chapel Hill, Chapel Hill, NC, United States; ^3^ Center for Gastrointestinal Biology and Disease, The University of North Carolina at Chapel Hill, Chapel Hill, NC, United States; ^4^ Lineberger Comprehensive Cancer Center, The University of North Carolina at Chapel Hill, Chapel Hill, NC, United States

**Keywords:** inflammatory bowel disease, colorectal cancer, microbiota, *Escherichia coli*, metabolites, inflammation, metal, therapeutics

## Abstract

Inflammatory bowel disease (IBD) is a significant global health problem that involves chronic intestinal inflammation and can involve severe comorbidities, including intestinal fibrosis and inflammation-associated colorectal cancer (CRC). Disease-associated alterations to the intestinal microbiota often include fecal enrichment of *Enterobacteriaceae*, which are strongly implicated in IBD development. This dysbiosis of intestinal flora accompanies changes in microbial metabolites, shaping host:microbe interactions and disease risk. While there have been numerous studies linking specific bacterial taxa with IBD development, our understanding of microbial function in the context of IBD is limited. Several classes of microbial metabolites have been directly implicated in IBD disease progression, including bacterial siderophores and genotoxins. Yet, our microbiota still harbors thousands of uncharacterized microbial products. In-depth discovery and characterization of disease-associated microbial metabolites is necessary to target these products in IBD treatment strategies. Towards improving our understanding of microbiota metabolites in IBD, it is important to recognize how host relevant factors influence microbiota function. For example, changes in host inflammation status, metal availability, interbacterial community structure, and xenobiotics all play an important role in shaping gut microbial ecology. In this minireview, we outline how each of these factors influences gut microbial function, with a specific focus on IBD-associated *Enterobacteriaceae* metabolites. Importantly, we discuss how altering the intestinal microenvironment could improve the treatment of intestinal inflammation and associated disorders, like intestinal fibrosis and CRC.

## Introduction

Inflammatory bowel disease (IBD) is a global health concern, characterized by chronic and severe inflammation of the gastrointestinal (GI) tract. Though the etiology of IBD is poorly understood, we know that the immunological responses underlying IBD development are governed by a combination of environmental and genetic factors (Gevers et al., 2014; Sartor and Wu, 2017; Imhann et al., 2018). Important genetic modifications affecting IBD are mostly connected with epithelial barrier function and the immunological pathways involved in the recognition and elimination of microorganisms (Consortium et al., 2015; McGovern et al., 2015). Disrupting the balance between gut mucosal tissues and the microbiota shapes host immune responses, influencing IBD development (Lavelle and Sokol, 2020).

Decades of research with human subjects and animal models of IBD correlate certain bacterial taxa and loss of microbial diversity with IBD (Taurog et al., 1994; Darfeuille-Michaud et al., 1998; Eckburg et al., 2005; Frank et al., 2007; Gevers et al., 2014). Several studies indicate that IBD patients versus healthy subjects have a loss of protective anaerobes and an increased abundance of potentially pathogenic species in their gut microbiota (Alhagamhmad et al., 2016). However, recent investigations reveal that microbial function, rather than composition, is more consistently perturbed in the IBD gut (Gevers et al., 2014; Franzosa et al., 2019). Franzosa *et al*. identified putative mechanistic associations between the IBD microbiome and its metabolites using a multi-omics approach (Franzosa et al., 2019). Despite identifying hundreds of differentially abundant taxa, metabolites, and associations within, only 43% of detected metabolites could be assigned to molecular classes based on the Human Metabolome Database (HMDB) (Donia and Fischbach, 2015; Franzosa et al., 2019). This study demonstrates how little we know about the composition of the human gut metabolome. Thus, to better understand the role of the gut metabolome in disease, we need to define the factors that shift microbial function.

Our microbiota consists of hundreds of bacterial species, which produce thousands of unique small molecules and secondary metabolites (Donia et al., 2014; Donia and Fischbach, 2015). Several classes of microbial metabolites are the focus of intense research, including bile acid derivatives, short-chain fatty acids (SCFA), and tryptophan metabolites. In addition, microbial composition and the production of microbial metabolites can be affected by the diet, which is widely considered a risk factor for the development of IBD (Mentella et al., 2020; Sugihara and Kamada, 2021). During symbiosis, gut resident bacteria use diet-derived nutrients for growth and colonization, and both the host and microbiota use microbial metabolites to maintain intestinal homeostasis. However, intake of certain components, such as high fat and low fiber, induce gut dysbiosis and promote intestinal inflammation (Lee et al., 2015; Makki et al., 2018; Kolodziejczyk et al., 2019; Zmora et al., 2019; Leeming et al., 2021; Tanes et al., 2021). Further study is still needed to understand the complex relationship between diet, metabolites, and intestinal homeostasis, which will aid in the prognosis and treatment of IBD at a personalized level (Aldars-García et al., 2021).

In this mini review, we examine host relevant factors that alter the pool of microbiota-derived metabolites. *Enterobacteriaceae*, notably adherent-invasive *Escherichia coli* (AIEC), are abundant in IBD patients and are implicated in disease progression (Darfeuille-Michaud et al., 1998; Kim et al., 2005; Arthur et al., 2012; Elhenawy et al., 2019; Ellermann et al., 2019). Thus, we specifically discuss *Enterobacteriaceae*-derived metabolites, including those implicated in IBD-associated intestinal fibrosis and colorectal cancer (CRC). Providing an improved understanding of microbial metabolite regulation will ultimately improve treatment for IBD and IBD-associated diseases.

## Inflammation

Aberrant immune responses to resident microbes, in the context of environmental factors and genetic susceptibility, shape the IBD patient microbiome. Common features of the inflammation-associated microbiome include loss of diversity and a bloom of *Enterobacteriaceae* at the mucosa, which permits the close association of microbes with the host epithelium (Darfeuille-Michaud et al., 1998; Schultsz* et al., 1999; Martin et al., 2004; Baumgart et al., 2007; Johansson et al., 2014; Sartor and Wu, 2017). Through interactions with epithelial and immune cells of the intestine, the IBD-associated microbiota and its metabolites alter the balance of immunoregulatory and effector immune responses, which further fuel this inflammatory environment and induce functional changes in the bacterial community (Sartor and Wu, 2017). Notably, *E. coli* at the inflamed mucosal interface have an altered transcriptome, which likely contributes to disease progression (Patwa et al., 2011; Tchaptchet et al., 2013; Arthur et al., 2014). Namely, *E. coli* stress response genes *gadA* and *gadB*, *ibpA* and *ibpB*, are upregulated in response to chronic intestinal inflammation in the interleukin-10 deficient (*Il10^-/-^*) IBD mouse model and pro-carcinogenic *E. coli pks* genes are upregulated in response to IBD-associated cancer in azoxymethane (AOM)/*Il10^-/-^* mice (Patwa et al., 2011; Tchaptchet et al., 2013; Arthur et al., 2014).

### Oxygenation

Complex nutrient utilization strategies highlight the diverse functional capacity of the microbiota. For example, host consumption of a high-fat diet alters host colonocyte and microbial metabolism, stimulating the growth of IBD-associated intestinal *E. coli* (Turnbaugh et al., 2009; Litvak et al., 2018). Complex host:microbe interactions drive this phenomenon and have been well-reviewed (Winter and Bäumler, 2014; Litvak et al., 2017; Litvak et al., 2018; Shelton and Byndloss, 2020). Briefly, loss of butyrate-producing microbes in the inflamed gut reduces activation of butyrate sensor PPARγ in colonocytes, which prevents beta-oxidation and increases bioavailable oxygen (Byndloss et al., 2017). Epithelial oxygenation drives aerobic respiration of pathogenic and non-pathogenic *Enterobacteriaceae* and fuels their expansion at the mucosa (Litvak et al., 2017; Cevallos et al., 2019; Chanin et al., 2020). Currently, the impact of gut oxygenation and/or inflammation-associated loss of anaerobiosis on *E. coli* metabolite production has not been explored.

### Mucin Degradation

The flexibility of *Enterobacteriaceae* metabolism and carbon utilization are implicated in its ability to persist in the inflamed gut and promote inflammatory disease (Zhang et al., 2022). Undigested carbohydrates and complex sugars in the intestinal mucus layer serve as an important energy source for gut microbes, including AIEC (Kim et al., 2017). In fact, comparative genomics and functional assays have revealed that the metabolism of fucose, 1,2-propanediol, ethanolamine, and downstream metabolites present in the gut are important for AIEC persistence in the inflamed GI tract (Dogan et al., 2014; Delmas et al., 2019; Elhenawy et al., 2019; Ormsby et al., 2019; Viladomiu et al., 2021). *E. coli* from the stool of IBD patients often express genes for metabolism of ethanolamine (*eutB, eutS*) and 1,2-propanediol (*pduC*), which are thought to be a metabolic adaptation that is advantageous for AIEC to overcome nutritional limitation and colonize the gut (Ormsby et al., 2019; Viladomiu et al., 2021). However, there is still a lot to uncover and understand on how the metabolism of these substrates impact *Enterobacteriaceae* metabolite production and pro-inflammatory capacity.

## Metal Availability

The bioavailability of metals in the gut is tightly controlled, especially during states of inflammation. Changes in metal availability are strongly linked to bacterial virulence and persistence in IBD. *Enterobacteriaceae* produce metabolites that can overcome host metal limitation and contribute to disease progression.

### Nutritional Immunity

During inflammation, the host limits nutrient availability to restrict growth of invading microbes (Cassat and Skaar, 2013; Becker and Skaar, 2014). Bioavailability of trace metals (e.g., iron, zinc, and copper) are tightly controlled and can change based on host diet and inflammation status (Pajarillo et al., 2021). Metals are vital nutrients for all forms of life and act as cofactors for essential proteins (Andreini et al., 2008; Hood and Skaar, 2012; Diaz-Ochoa et al., 2014; Palmer and Skaar, 2015). Thus, throughout evolution, the battle for metal acquisition has persisted between bacteria and host (Hood and Skaar, 2012; Skaar and Raffatellu, 2015). Hosts developed “Nutritional Immunity” to overcome sequestration of essential metals by invading pathogens, especially by controlling metal concentration through metal-binding proteins such as iron-binding lactoferrin, transferrin, lipocalin, and zinc-binding calprotectin (Oram and Reiter, 1968; Zackular et al., 2015; Palmer and Skaar, 2016). However, microbes also developed methods for effectively sequestering host metals, even during nutritional immunity. One means of evading nutritional immunity is *via* “stealth siderophores” which cannot be neutralized by the host.

### Siderophores

In metal-limited environments, like the inflamed gut, bacteria produce siderophores to outcompete the host for essential metals (Ellermann and Arthur, 2017). Siderophores are low molecular weight compounds that bind various metals with high affinity (Hider and Kong, 2010; Holden and Bachman, 2015). The structural variety of siderophores makes it easy for bacteria to acquire essential metals and overcome growth limitations within the host (Hider and Kong, 2010; Holden and Bachman, 2015).

*Enterobacteriaceae* produce a siderophore called Enterobactin (Ent), which has an extremely high binding affinity to ferric iron  (Pollack and Neilands, 1970; Young and Gibson, 1979; Raymond et al., 2003). Ent is commonly produced in low iron environments through de-repression of the Ent biosynthetic gene cluster (BGC) by the DNA-binding Ferric Uptake Regulation protein, Fur (Crosa and Walsh, 2002). Host neutrophils and epithelial cells secrete lipocalin-2 (Lcn2) into the mucosa, which binds the Ent-iron complex and inhibits the growth of pathogenic bacteria (Flo et al., 2004; Bachman et al., 2012). Interestingly, Ent produced by commensal bacteria plays an important role in mitochondria iron uptake and homeostasis, which has a beneficial effect on host development (Qi and Han, 2018). Therefore, although siderophores have been thought to play a negative role in iron homeostasis and induce pathogenesis, Ent could provide a host advantage during colonization with certain bacterial strains.

Yersiniabactin (Ybt) is a stealth siderophore, harbored by some host-associated *E. coli* strains, that evades host nutritional immunity. The Ybt BGC, like Ent, is repressed by Fur in the presence of ample iron (Haag et al., 1993; Gehring et al., 1998; Pelludat et al., 1998; Crosa and Walsh, 2002). Ybt was originally described as an essential fitness factor for *Yersinia pestis* and is common in *Klebsiella* spp. and gut *E. coli* (Bach et al., 2000; Lawlor et al., 2007; Fetherston et al., 2010; Dogan et al., 2014; Brumbaugh et al., 2015; Ellermann et al., 2019). Ybt-mediated iron acquisition is important for virulence, niche formation by pathogens, and colonization resistance by commensal microbes (Koh et al., 2015; Ellermann and Arthur, 2017). Ybt uniquely binds biologically important metals other than iron to aid in infection, including zinc, copper, and nickel (Chaturvedi et al., 2012; Bobrov et al., 2014; Koh et al., 2015; Robinson et al., 2018) A recent paper demonstrated that Ybt-zinc acquisition allows the probiotic *E. coli* Nissle to tolerate host-mediated zinc sequestration, allowing beneficial Nissle to bloom in the gut (Behnsen et al., 2021). However, it is reasonable to suspect that Ybt also permits AIEC expansion and promotes further inflammation in the gut of IBD patients. In addition, Ybt can steal metal from host cells, disrupting host metal homeostasis and promoting disease. We demonstrated that AIEC production of Ybt, but not bacterial utilization of this siderophore, enhanced inflammation and induced intestinal fibrosis in the *Il10^-/-^* mouse model (Ellermann et al., 2019). This study suggests that Ybt has direct effects on the host. Indeed, Ybt from *Klebsiella* spp. can deplete iron from airway epithelial cells and activate the hypoxia-inducible factor 1-alpha (HIF-1α) pathway (Holden et al., 2014). But how Ybt modulates host HIF-1α activation, and the biological consequences of this regulation remain unclear.

## Interbacterial Community Functions

Social cooperation and resource capture can protect bacteria against antimicrobials and negatively affect the host immune response. This is exemplified in biofilm communities. AIEC biofilms promote survival in macrophages, an AIEC-defining feature linked to their enhanced inflammatory potential (Martinez-Medina et al., 2009a; Martinez-Medina et al., 2009b; Martinez-Medina and Garcia-Gil, 2014; Ellermann et al., 2015; Renouf et al., 2018; Prudent et al., 2021). Additionally, *E. coli* biofilms are abundant at the mucosa of IBD patients, and genes required for biofilm component biosynthesis are upregulated in IBD patient microbiomes (Swidsinski et al., 2005; Renouf et al., 2018). Here we discuss how social interactions, such as interbacterial competition or functional synergy, shape the disease-causing potential of our microbiota.

### Interbacterial Competition

Intestinal *E. coli* compete for nutrients and niches in the intestine in a variety of ways, including Type VI secretion systems (T6SSs), siderophores, and microcins (Sorbara and Pamer, 2019). Microcins are small, secreted proteins with antimicrobial activities that can kill related bacteria by binding to siderophores and entering the cell like a “Trojan Horse” (Duquesne et al., 2007; Massip and Oswald, 2020). Microcins are produced by some uropathogenic, intestinal, and probiotic *Enterobacteriaceae* and may be exploited as therapeutics to prevent the bloom and persistent colonization of pathogens and inflammation-associated *E. coli* (Sassone-Corsi et al., 2016). While this area of research is understudied in the IBD field, the importance of microcins in infectious disease colonization resistance and their functional relationship with siderophores makes them an interesting target to pursue for microbiome modulation.

Colibactin is a microbially-derived genotoxin produced by a BGC known as the *pks* island. It is well appreciated that colibactin elicits host DNA damage and is implicated in the initiation of CRC (Nougayrède et al., 2006; Arthur et al., 2012; Arthur et al., 2014; Arthur, 2020; Dziubańska-Kusibab et al., 2020; Lopez et al., 2020; Pleguezuelos-Manzano et al., 2020). However, colibactin is a complex natural product with a high biosynthetic cost and no clear role in bacterial fitness, beyond the newly discovered role for the ClbS immunity protein that protects bacteria from colibactin-triggered prophage induction (Silpe et al., 2022). Although poorly understood, colibactin regulation is linked to *E. coli* virulence factors, including the Ent and Ybt siderophores, low iron, and antibiotic stress (Martin et al., 2017; Sadecki et al., 2021). Although mechanisms remain unclear, it has been postulated that colibactin protects against membrane stress and may have antibiotic activity against competing microbes. Sugimoto *et al*. developed a module to discover BGCs in metagenomic sequencing data of the human microbiome and revealed BGCs that encode 13 type II polyketides; among them, two polyketides have strong antibacterial activities (Sugimoto et al., 2019). This study suggests that uncharacterized BGC products may be important contributors to microbe:microbe competition in the gut.

### Microbiota Functional Synergy

Opposite of bacterial antagonism, neighboring microbes can promote functional synergy within polymicrobial communities. This can be detrimental in the context of host disease, as the microbiota can inadvertently heighten the virulence activity of pathogens. For example, *Bacteroides thetaiotaomicron* can alter gut mucins, enhancing *E. coli* virulence (Becattini et al., 2016). In addition, *Enterococcus faecalis* stimulates *E. coli* siderophore production that promotes *E. coli* growth and biofilm formation in metal-limited environments (Keogh et al., 2016). Propionate, an abundant SCFA commonly derived from *Bacteroides* spp., can mediate colonization resistance against *Salmonella enterica* serovar Typhimurium (*S. Tm*) (Shelton et al., 2022). However, in the inflamed gut, both *S. Tm* and *E. coli* use nitrate and nitrate-dependent propionate metabolism for intestinal expansion (Winter et al., 2013; Shelton et al., 2022) Furthermore, *S. Tm* and pathogenic *E. coli* utilize aspartate ammonia-lyase (*aspA*)*-*dependent fumarate respiration for growth in the murine gut during colitis (Yoo et al., 2022). Numerous instances of microbial functional synergy have been described in the inflamed gut (Hughes et al., 2017; Spiga et al., 2017; Gillis et al., 2018; Zhu et al., 2020), and it will be important to understand their contribution to disease in human IBD patients.

## Xenobiotics

Numerous classes of xenobiotics (i.e., pharmaceuticals) are metabolized by single isolates and communities of the gut microbiome (Javdan et al., 2020). As such, drug-drug-microbiota interactions vary between individuals. Recently developed frameworks to evaluate drug-microbiota-metabolome interactions are expected to revolutionize personalized medicine (Spanogiannopoulos et al., 2016; Javdan et al., 2020; Quinn et al., 2020). There are at least 108 pharmacological drugs metabolized by microbes, including IBD patient anti-inflammatories and cancer therapeutics. Notable drugs chemically transformed by gut microbial enzymes include anti-inflammatory methotrexate and anti-cancer drugs, irinotecan, CPT-11, and gemcitabine (Wallace et al., 2010; Geller et al., 2017; Bhatt et al., 2020; Nayak et al., 2021), cardiac drug digoxin (Haiser et al., 2014), and antidiabetic acarbose (Balaich et al., 2021). However, xenobiotics and their chemically transformed metabolites can also directly target bacteria, influencing their virulence and metabolite production. Microbiota drug biomodulation can influence treatment efficacy efficacy and produce undesirable undesirable side effects.

### IBD Therapeutics

IBD anti-inflammatories, such as anti-tumor necrosis factor-alpha (TNFα), improve IBD disease activity and can modulate the microbiome in a way that reduces the development of IBD-associated CRC (Yang et al., 2020). This reduction in carcinogenicity of the microbiome is attributed to decreased abundance of colibactin-producing *E. coli* (Yang et al., 2020). Yet, it is poorly understood how colibactin production is modulated on a per-cell basis and whether xenobiotics can impact colibactin production and pro-carcinogenic effects.

Pharmaceuticals can be used as building blocks for bacterial metabolite production by bacteria. *E. coli* use an enzyme termed YbtS, a salicylate synthase, in the early steps of building Ybt. It is possible that exogenous salicylate (i.e., Asprin) can compensate for the loss of this enzyme and, even in its absence, promote Ybt production (Pelludat et al., 2003). Salicylates also alter bacterial susceptibility to antibiotics by activating *E. coli* multiple antibiotic resistance (Mar), leading to increased expression of the multidrug efflux pump AcrAB-TolC (Cohen et al., 1993; Sharma et al., 2017). TolC is also important in Ent efflux, so it will be valuable to assess how salicylates can impact bacterial fitness and pro-inflammatory potential in the gut.

Another salicylate-derived drug, mesalamine (5-aminosalicylic acid, 5-ASA), is an NSAID used in the treatment of IBD and specifically ulcerative colitis (Campieri et al., 1981; Dew et al., 1983). Mesalamine reduces microbial burden in the feces and at the mucosal niche, impacting AIEC virulence gene expression, motility, epithelial adherence/invasion, and intra-macrophage survival – all believed to help control active disease symptoms (Zhang et al., 2018; Dai et al., 2021). These direct effects of mesalamine on bacteria may be driven by the inhibition of polyphosphate kinase and rapid loss of bacterial polyphosphate levels, which can sensitize bacteria to oxidative stress and reduce colonization and biofilm formation in inflamed environments (Dahl et al., 2017). Mesalamine treatment of colibactin-producing *E. coli* can decrease transcription of the colibactin *pks* island, but this appears to be independent of the polyphosphate kinase, indicating a novel means of regulation (Zhang et al., 2018; Tang-Fichaux et al., 2020). Direct effects on gene expression also occur in *S. Tm*, where mesalamine impacts genes associated with bacterial invasion, cellular metabolism, and stress response (Kaufman et al., 2009). Furthermore, subinhibitory levels of the antibiotic sulfamethoxazole (SMX) induce *E. coli* metabolite production of antioxidants termed colipterins, which induce host anti-inflammatory IL-10 and reduce colitis in mouse models (Park et al., 2020). It will be important to determine how these clinically relevant IBD drugs impact *E. coli* behavior and metabolite production among the rich consortium of other microbes and both host and microbe-derived metabolites within the inflamed gut.

### Antibiotics

Modern-day antibiotics are invaluable in controlling pathogenic infections but can have long-lasting effects on the microbiota, including antibiotic resistance among resident gut microbes. Resistance mechanisms develop in natural environments and can easily diffuse through microbial populations (Becattini et al., 2016). In addition, large-scale antibiotic pressure through medical and industrial practices has led to a surge of antibiotic resistance. Based on a study of healthy subjects, it was estimated that 20% of bacteria isolated from feces harbored antibiotic resistance elements (Sommer et al., 2009). Another study found a greater incidence of antibiotic resistance among *E. coli* cultured from the ileum of IBD and non-IBD patients, most notably to antibiotics commonly used to treat GI infection (Dogan et al., 2013). Antibiotic resistance mechanisms often cause a shift in bacterial metabolism; thus, it is common for the downregulation of energy-intensive microbial activities to be associated with antibiotic resistance. We recently observed that evolved resistance of *E. coli* to polymyxin B surprisingly upregulates colibactin production, including elevated transcription and protein levels, which induces increased DNA damage in mammalian cells (Sadecki et al., 2021). Mechanisms driving this effect remain unknown, yet the impact of IBD-associated antibiotic resistance on bacterial metabolite production is an active area of research, due to its likely importance in intestinal disease.

## Discussion

**Figure 1:**
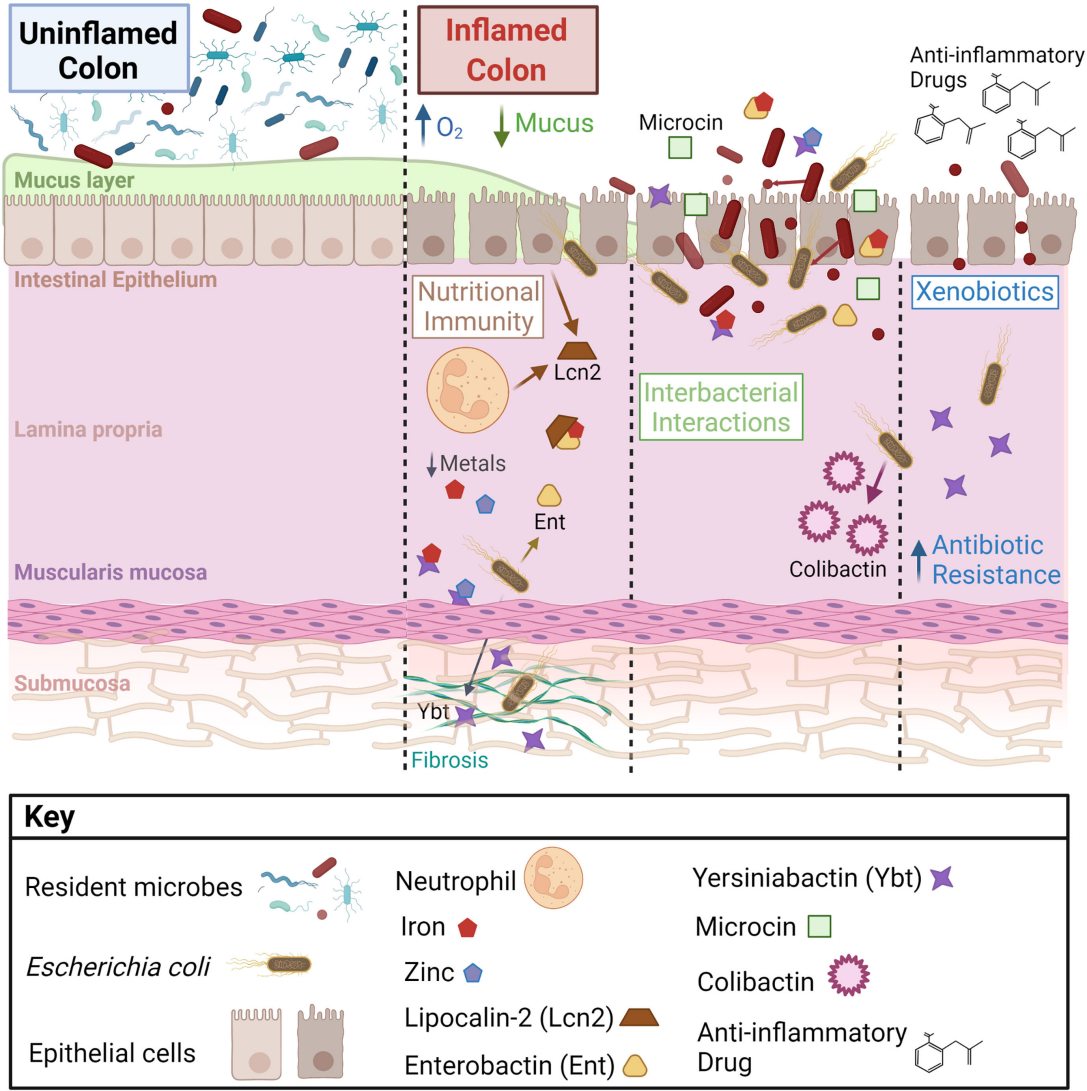
Intestinal microenvironmental stressors that influence bacterial metabolite production and disease on the host. During inflammation, increased epithelial oxygenation and mucus degredation is linked to a loss of gut microbial diversity and enrichment of *Enterobacteriaceae*, notably *Escherichia coli*. *E. coli* at the mucosal interface and within intestinal tissue respond to microenvironmental changes, which alters their disease-causing potential. Important factors affecting *E. coli* metabolite production in the gut include host inflammation, nutritional immunity, interbacterial interactions, and exogenous pharmaceuticals.

During IBD, a pro-inflammatory state imposes pressure that shifts microbiome function, damaging gut tissue integrity, and resulting in disease (Caruso et al., 2020). Many factors impact the composition and function of this “dysbiotic” microbiota, including bacterial and host metal metabolism (**Figure 1**). The impact of host metal-limiting proteins and the relative contribution of bacterial virulence versus host metal homeostasis remain to be explored in the context of chronic inflammatory disease. Altering the bioavailability of essential metals, co-factors for many microbial and host enzymes, is likely to influence chronic disease through effects on both host and microbes (Chaturvedi et al., 2012; Holden et al., 2014; Koh et al., 2015; Robinson et al., 2018; Ellermann et al., 2019). In the gut, members of the microbiota defend themselves by utilizing beneficial molecules and producing inhibitory molecules during interbacterial competition that may influence host intestinal disease. Recent reports identified that *Enterobacteriaceae* can overcome colonization resistance using SCFAs, alternative electron acceptors, and carbon sources (Yoo et al., 2021; Shelton et al., 2022) However, *Enterobacteriaceae* harbor such a large and diverse accessory genome that many mechanisms of microbial competition and persistence in the gut remain unknown. Studying a diverse repertoire of gut *Enterobacteriaceae* associated with health and chronic intestinal disease is likely to reveal novel mechanisms and unique metabolites important for thriving in the inflamed gut and driving inflammation-associated chronic intestinal disease.

There remains a large pool of un-sequenced microbes and uncharacterized microbial genes in the human gut microbiome. Discovery and in-depth study of microbiota metabolites requires high-throughput interdisciplinary approaches. Promising studies utilizing targeted and untargeted multi-omics approaches have yielded key findings on microbiota metabolites in disease (Sugimoto et al., 2019; Lavelle and Sokol, 2020). In tandem with large-scale studies using prospective cohorts, new pipelines and frameworks for meta-analyses of available datasets are rapidly improving our understanding of metabolite-driven host:microbe and microbe:microbe interactions during inflammatory disease. Importantly, host-associated microbial metabolites could be used as novel biomarkers for disease and provide mechanistic insight into host:microbe interactions driving IBD and IBD-associated pathologies. Mapping patient microbial metabolite profiles in relation to community structure analysis, host genetics, and clinical disease features could be a powerful means to predict and monitor a patient’s disease course.

To better study the causal role of microbial metabolites in IBD and IBD-associated complications, we need to carefully model the complex host intestinal environment. One constraint to building better model systems is our limited understanding of the mucosal niche where most host:microbe interactions occur. Recent technologies may help breach this gap by co-localizing microbial species and their metabolites in the host environment (Garg et al., 2017). However, to demonstrate the causality of microbes and their products, we need to build better longitudinal clinical studies. When designing these studies, we should especially consider strain-level variations and how changes in environmental factors shape microbiota-mediated effects on the host (Lam et al., 2019). Broad-spectrum antibiotics or inhibitors could have negative consequences on commensal microbes. Therefore, it is vital to precisely manipulate the microbial targets implicated in disease.

One approach for targeted microbiota management includes modifying patient-associated resident bacterial strains. Ways to selectively target microbial strains include bacteriophage therapy, which has demonstrated efficacy against bacterial biofilms and persister cells (Lewis, 2013; Lam et al., 2019). Also, microbiota editing could be performed by targeting unique microbial features. For example, oral tungsten was used to prevent *E. coli* expansion and reduce intestinal inflammation (Zhu et al., 2018). In addition, anti-adhesive molecules have been proposed to limit AIEC intestinal colonization (Schirmer et al., 2019). Another option for targeted treatment is to specifically inhibit key metabolite functions. As proof-of-principle, a specific inhibitor of bacterial β-glucuronidases was found to improve GI toxicity caused by biomodulation of a chemotherapeutic drug (Bhatt et al., 2020). Specialized diets could also be an indirect method for modulating gut microbiota function. The unnecessary use of antibiotics to control microbiota function should be avoided. However, if depleted commensals are an effect of treatment, it may be beneficial to regain community balance through fecal microbiota transplant (FMT) or administration of a known bacterial consortium (Schirmer et al., 2019). Notably, these classes affect the host through various mechanisms and illuminate the connections between the host, a dysbiotic microbiota, and an altered metabolite milieu.

## Author Contributions

Most of the text was written by LL and JA. The section on metal availability was written and additional text edited by J-HA. The figure was created by LL and TA. All authors contributed to the article and approved the submitted version.

## Funding

Funding is from R01 DK124617 and R21 AI159786 (JA). LL is a member of the Royster society of fellows. TA holds a Fulbright/CAPES scholarship.

## Conflict of Interest

The authors declare that the research was conducted in the absence of any commercial or financial relationships that could be construed as a potential conflict of interest.

## Publisher’s Note

All claims expressed in this article are solely those of the authors and do not necessarily represent those of their affiliated organizations, or those of the publisher, the editors and the reviewers. Any product that may be evaluated in this article, or claim that may be made by its manufacturer, is not guaranteed or endorsed by the publisher.
